# Supplementary material for: Land management shapes drought responses of dominant soil microbial taxa across grasslands
Source: Nat Commun. 2024 Jan 2;15:29. doi: 10.1038/s41467-023-43864-1 (PMC10762234; doi:10.1038/s41467-023-43864-1)
Supplement: Supplementary file 1 — Supplementary Information [file 41467_2023_43864_MOESM1_ESM.pdf]

SUPPLEMENTARY INFORMATION

Land management shapes drought responses of dominant soil microbial taxa across grasslands

Lavallee, J. M.[1,2], Chomel, M.[1,3], Alvarez Segura, N.[4,5], de Castro, F.[6,7], Goodall, T.[8], Magilton, M.[6,9], Rhymes, J. M.[1,10], Delgado-Baquerizo, M.[11,12], Griffiths, R. I.[8,13], Baggs, E. M.[14], Caruso, T.[15], de Vries, F. T.[1,16], Emmerson, M.[6], Johnson, D.[1], Bardgett, R. D.[1]

Affiliations

1. Department of Earth and Environmental Sciences, The University of Manchester, Oxford Road, Manchester M13 9PT, UK
2. Environmental Defense Fund, 257 Park Ave S, New York, NY, 10010 USA
3. FiBL France, Research Institute of Organic Agriculture, 26400 Eurre, France
4. Institute of Biological and Environmental Sciences, University of Aberdeen, St Machar Dr, Old Aberdeen, Aberdeen AB24 3UL, UK
5. EURECAT – Centre Tecnològic de Catalunya, C/ de Bilbao, 72, 08005 Barcelona, Spain
6. School of Biological Sciences and Institute for Global Food Security, Queen's University of Belfast, 19 Chlorine Gardens, Belfast BT9 5DL, UK
7. AgriFood & Biosciences Institute, 18a Newforge Ln, Belfast BT9 5PX, UK
8. UK Centre for Ecology & Hydrology Wallingford, Maclean Building, Benson Lane, Crowmarsh Gifford, Wallingford, Oxfordshire OX10 8BB, UK
9. School of Life Sciences, University of Lincoln, Brayford Pool, Lincoln LN6 7TS, UK
10. Centre for Ecology & Hydrology Bangor, Environment Centre Wales, Deiniol Road, Bangor LL57 2UW, UK
11. Laboratorio de Biodiversidad y Funcionamiento Ecosistémico. Instituto de Recursos Naturales y Agrobiología de Sevilla (IRNAS), CSIC, Av. Reina Mercedes 10, E-41012, Sevilla, Spain
12. Unidad Asociada CSIC-UPO (BioFun). Universidad Pablo de Olavide, 41013 Sevilla, Spain
13. School of Natural Sciences, Bangor University, Deiniol Rd, Bangor LL57 2UR, UK
14. Global Academy of Agriculture and Food Systems, Royal (Dick) School of Veterinary Studies, University of Edinburgh, Midlothian EH25 9RG, UK
15. School of Biology and Environmental Science, University College Dublin, Dublin, Ireland
16. Institute for Biodiversity and Ecosystem Dynamics, University of Amsterdam, 1012 WX Amsterdam, Netherlands

* Corresponding Author: Jocelyn M. Lavallee, jlavallee@edf.org

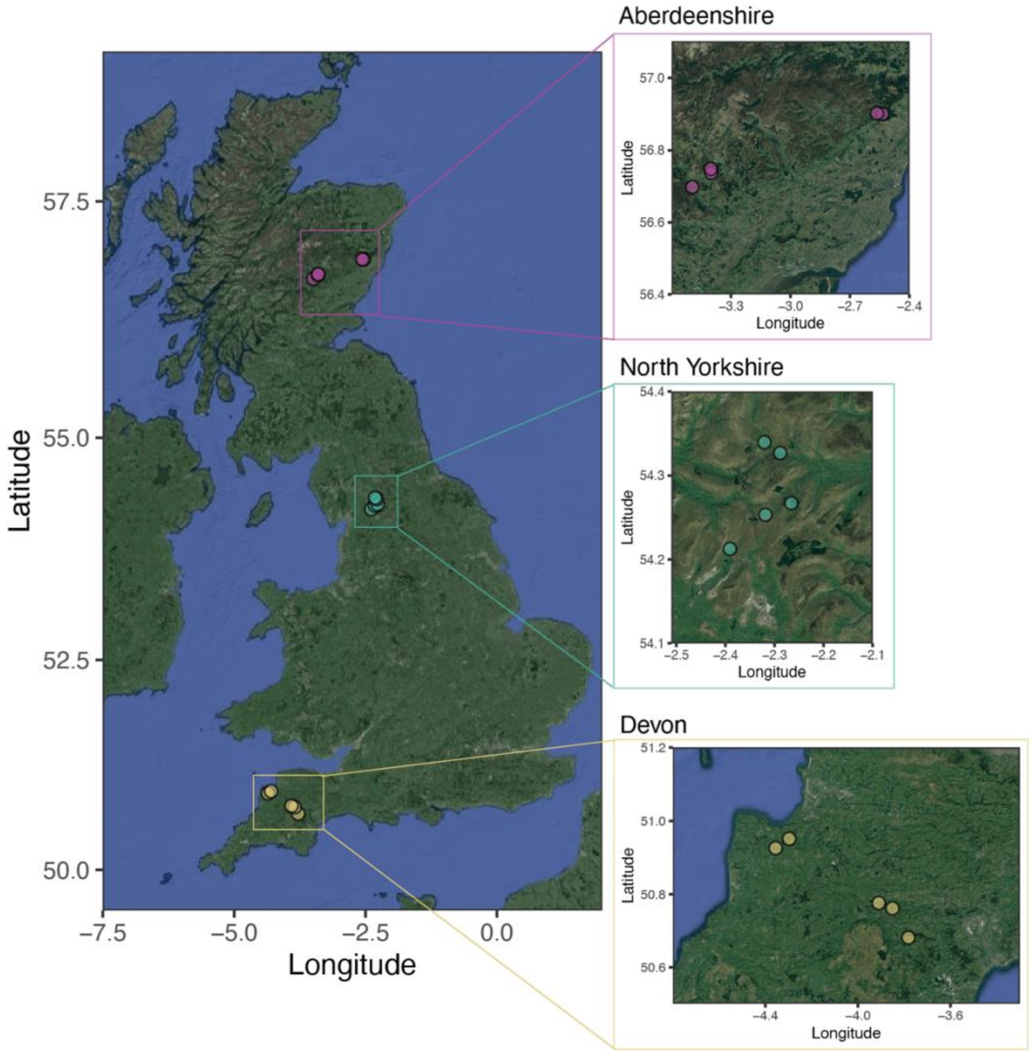

Supplementary Fig. 1. **Map of site locations**. Detail provided for each of the three regions (Devon, North Yorkshire, Aberdeenshire). Each point represents one site consisting of two paired fields with contrasting management (extensive, intensive).

Supplementary Table 1. **Characteristics of the grassland sites used in this study.** Each site consists of paired pastures under contrasting management ("extensive" or "intensive"). Paired of fields at each site are adjacent or located < 0.5 km apart.

**Region**	**Site**	**Intensive**					**Extensive**				
		Fertilisation	Lime application	Cuttings	Number of plant species	Soil bulk density	Fertilisation	Lime application	Cuttings	Number of plant species	Soil bulk density
Devon	1	Synthetic NPK	Yes	1-2 times per year	≥ 5	0.65	Unfertilized for ≥ 15 years	None	None	≥ 9	0.53
	2	Synthetic NPK and manure slurry	3 tonnes $acre^{-1}$ applied in 2010	2 times per year	≥ 5	0.65	Unfertilized for ≥50 years	None	None	≥ 15	0.61
	3	Synthetic NPK	Yes	Once per year	≥ 5	0.94	Unfertilized for ≥ 20 years	None	Once per year	≥ 13	0.88
	4	Synthetic NPK (100 kg 20-10-10 $acre^{-1}$ $year^{-1}$)	Yes	1-2 times per year	≥ 7	0.76	Unfertilized for ≥ 20 years	None	None	≥ 13	0.63
	5	Manure slurry	2 tonnes $acre^{-1}$ applied in 2009	3 times per year	≥ 6	0.84	Unfertilized for ≥10 years	None	None	≥ 9	0.61
North York-shire	1	Synthetic NPK (50 kg 25-5-5 $acre^{-1}$ $year^{-1}$) and manure	Yes	Not in past 17 years	≥ 9	0.60	Unfertilized for ≥ 15 years	None	None	≥ 9	0.68
	2	Synthetic NPK and manure	1 tonne $acre^{-1}$ every 3 years	Once per year	≥ 9	0.34	Unfertilized for ≥ 65 years	None	None	≥ 15	0.31
	3	Synthetic NPK (50 kg 25-5-5 $acre^{-1}$ $year^{-1}$) and manure	2 tonnes $acre^{-1}$ every 7 years	Once per year	≥ 11	0.44	Unfertilized for ≥ 50 years	None	None	≥ 11	0.60
	4	Synthetic NPK and manure	Yes	Once per year	≥ 8	0.33	Unfertilized for ≥ 10 years	None	None	≥ 10	0.65
	5	Synthetic NPK (75 kg 25-5-5 $acre^{-1}$	Once per year	Once per year	≥ 8	0.53	Unfertilized for ≥ 17 years	None	None	≥ 9	0.18

		year^{-1}) and manure									
Aberdeen-shire	1	Synthetic NPK (75 kg 24-0-0 acre^{-1} year^{-1}, P-K some years)	1.6 tonnes acre^{-1} in 2012	Once per year	≥ 10	0.61	Unfertilized for ≥ 30 years	None	None	≥ 15	0.50
	2	Synthetic NPK (100 kg 25-5-5 acre^{-1} year^{-1})	2 tonnes acre^{-1} every 6 years	N/A	≥ 7	0.85	Unfertilized for ≥ 25 years	None	None	≥ 13	0.74
	3	Synthetic NPK (100 kg 25-5-5 acre^{-1} year^{-1}) and manure	2 tonnes acre^{-1} every 6 years	N/A	≥ 6	0.75	Unfertilized for ≥ 25 years	None	None	≥ 7	0.72
	4	Synthetic NPK or P-K until 2013	1.62 tonnes acre^{-1} in 2011	N/A	≥ 4	0.83	Unfertilized for ≥ 25 years	None	None	≥ 7	0.61
	5	Synthetic NPK or P-K until 2009	0.8 tonnes acre^{-1} in 2005	N/A	≥ 6	0.74	Unfertilized for ≥ 25 years	None	None	≥ 8	0.62

Supplementary Table 2. **Soil properties of study sites (control plots only).** Values are averaged across time points (n = 180 samples), except sand which was measured on composite samples at one time point (n = 30 samples). Ex = extensive, In = intensive.

Region	Site	Manage-ment	Total C (g C m^{-2})	Total N (g N m^{-2})	pH	Sand (%)	Fungi: bacteria ratio	Plant-available N (g N m^{-2})	Microbial biomass (g C m^{-2})	Soil temperature (°C)	Soil moisture (% vol)
**Devon**	**1**	**Ex**	7.6 ± 0.6	0.63 ± 0.05	5.6 ± 0.2	56.0	0.063 ± 0.037	1.1 ± 0.1	25.2 ± 2.0	18.9 ± 0.6	29.4 ± 1.5
		**In**	6.8 ± 1.6	0.56 ± 0.13	5.7 ± 0.1	68.5	0.039 ± 0.018	1.9 ± 0.5	23.6 ± 7.5	20.4 ± 0.8	44.3 ± 2.8
	**2**	**Ex**	3.4 ± 0.2	0.34 ± 0.02	5.8 ± 0.1	21.2	0.072 ± 0.016	0.6 ± 0.4	27.4 ± 8.6	16.7 ± 1.3	33.9 ± 19
		**In**	3.5 ± 0.3	0.36 ± 0.02	5.8 ± 0.1	21.5	0.053 ± 0.014	1.5 ± 0.9	28.6 ± 10.1	17.2 ± 0.2	31.1 ± 13.8
	**3**	**Ex**	3.8 ± 0.5	0.37 ± 0.04	5.6 ± 0.1	39.3	0.117 ± 0.042	2.2 ± 2.7	46.0 ± 11.2	18.3 ± 0.8	41.4 ± 10.3
		**In**	3.6 ± 0.5	0.36 ± 0.04	6.1 ± 0.2	53.0	0.053 ± 0.015	1.9 ± 0.6	20.8 ± 5.4	19.1 ± 0.6	34.8 ± 11
	**4**	**Ex**	3.2 ± 0.2	0.33 ± 0.02	5.3 ± 0.1	21.1	0.087 ± 0.041	1.0 ± 0.8	31.8 ± 14.0	17.6 ± 0.4	55.7 ± 9.7
		**In**	3.0 ± 0.2	0.33 ± 0.02	5.7 ± 0.2	28.2	0.065 ± 0.029	1.1 ± 0.6	28.1 ± 13.2	19.1 ± 1	51.6 ± 8.1
	**5**	**Ex**	3.4 ± 0.1	0.30 ± 0.01	5.3 ± 0.1	18.3	0.050 ± 0.016	0.3 ± 0.4	30.7 ± 7.0	21.3 ± 2.1	50.6 ± 7.9
		**In**	2.9 ± 0.8	0.28 ± 0.07	6.2 ± 0.2	23.0	0.065 ± 0.027	0.6 ± 0.8	31.7 ± 23.0	21.2 ± 3.6	51.2 ± 7.6
**North Yorkshire**	**1**	**Ex**	4.0 ± 0.4	0.38 ± 0.04	5.0 ± 0.2	32.5	0.039 ± 0.013	0.4 ± 0.2	19.4 ± 7.5	13.6 ± 2.8	66.3 ± 4
		**In**	4.1 ± 0.2	0.38 ± 0.02	5.4 ± 0.04	34.0	0.044 ± 0.021	1.9 ± 0.9	15.1 ± 2.8	15.3 ± 2.8	62.1 ± 2.8
	**2**	**Ex**	4.9 ± 0.9	0.32 ± 0.05	5.5 ± 0.2	48.2	0.037 ± 0.013	0.6 ± 0.3	41.4 ± 16.4	17 ± 3	65.7 ± 6.2
		**In**	5.8 ± 1.7	0.37 ± 0.11	5.5 ± 0.1	31.8	0.037 ± 0.011	0.8 ± 0.6	22.8 ± 6.4	15.9 ± 2.1	64.9 ± 2.8
	**3**	**Ex**	3.4 ± 0.8	0.29 ± 0.08	4.9 ± 0.5	33.9	0.086 ± 0.052	1.0 ± 0.4	28.6 ± 7.3	15 ± 2.4	55.3 ± 5.8
		**In**	3.0 ± 0.4	0.26 ± 0.04	4.7 ± 0.1	41.8	0.043 ± 0.026	2.4 ± 0.3	16.4 ± 5.3	13.8 ± 1.5	64.2 ± 2.9
	**4**	**Ex**	6.1 ± 2.7	0.41 ± 0.11	4.9 ± 0.3	39.5	0.059 ± 0.014	2.4 ± 0.8	26.0 ± 5.4	17.6 ± 2.8	36.2 ± 9
		**In**	7.0 ± 2.0	0.42 ± 0.13	4.8 ± 0.1	51.6	0.032 ± 0.007	3.3 ± 1.2	29.0 ± 13.3	17.2 ± 2.9	57.7 ± 8.8
	**5**	**Ex**	5.2 ± 2.7	0.36 ± 0.18	4.1 ± 0.1	56.2	0.040 ± 0.014	2.5 ± 0.8	21.1 ± 6.4	15.8 ± 2.8	65.7 ± 5.2
		**In**	6.0 ± 0.3	0.42 ± 0.03	4.7 ± 0.1	41.6	0.033 ± 0.004	3.4 ± 1.4	27.6 ± 6.4	14.9 ± 2.2	52.8 ± 9.6
**Aberdeen-shire**	**1**	**Ex**	3.0 ± 1.1	0.24 ± 0.09	5.1 ± 0.6	54.6	0.096 ± 0.024	2.1 ± 1.1	29.6 ± 5.3	19.8 ± 1.5	45.1 ± 2.8
		**In**	3.9 ± 1.0	0.33 ± 0.07	6.2 ± 0.1	58.5	0.036 ± 0.014	3.6 ± 2.2	27.8 ± 6.0	18.1 ± 3.1	50 ± 7.5
	**2**	**Ex**	7.3 ± 1.0	0.48 ± 0.06	4.6 ± 0.2	58.1	0.094 ± 0.027	1.5 ± 0.4	16.5 ± 4.1	16.7 ± 0.9	58.7 ± 2.5
		**In**	4.6 ± 1.1	0.34 ± 0.09	5.2 ± 0.04	41.9	0.049 ± 0.012	1.8 ± 1.3	11.0 ± 3.1	15.9 ± 0.8	61.3 ± 3.1
	**3**	**Ex**	6.9 ± 1.9	0.55 ± 0.16	4.5 ± 0.04	54.3	0.048 ± 0.015	3.0 ± 1.7	34.8 ± 8.8	19 ± 2.7	51.5 ± 2.9
		**In**	5.6 ± 1.6	0.48 ± 0.15	6.0 ± 0.4	47.6	0.036 ± 0.007	2.2 ± 1.4	20.6 ± 3.7	17.8 ± 2.5	55.7 ± 5.4
	**4**	**Ex**	7.0 ± 1.3	0.57 ± 0.09	4.4 ± 0.1	47.7	0.044 ± 0.013	2.9 ± 1.4	21.5 ± 3.4	19.1 ± 2	39.2 ± 3.7
		**In**	5.4 ± 0.6	0.43 ± 0.05	5.4 ± 0.1	42.4	0.066 ± 0.031	2.5 ± 1.4	9.3 ± 4.8	18.5 ± 1	52.5 ± 4.5
	**5**	**Ex**	6.9 ± 0.6	0.43 ± 0.04	4.4 ± 0.01	44.7	0.029 ± 0.009	2.2 ± 1.4	3.4 ± 0.9	19.2 ± 2.5	55.5 ± 7.3
		**In**	5.5 ± 1.7	0.43 ± 0.13	4.8 ± 0.05	41.3	0.046 ± 0.014	3.8 ± 1.3	24.0 ± 5.4	17.5 ± 1.7	54.2 ± 3.2

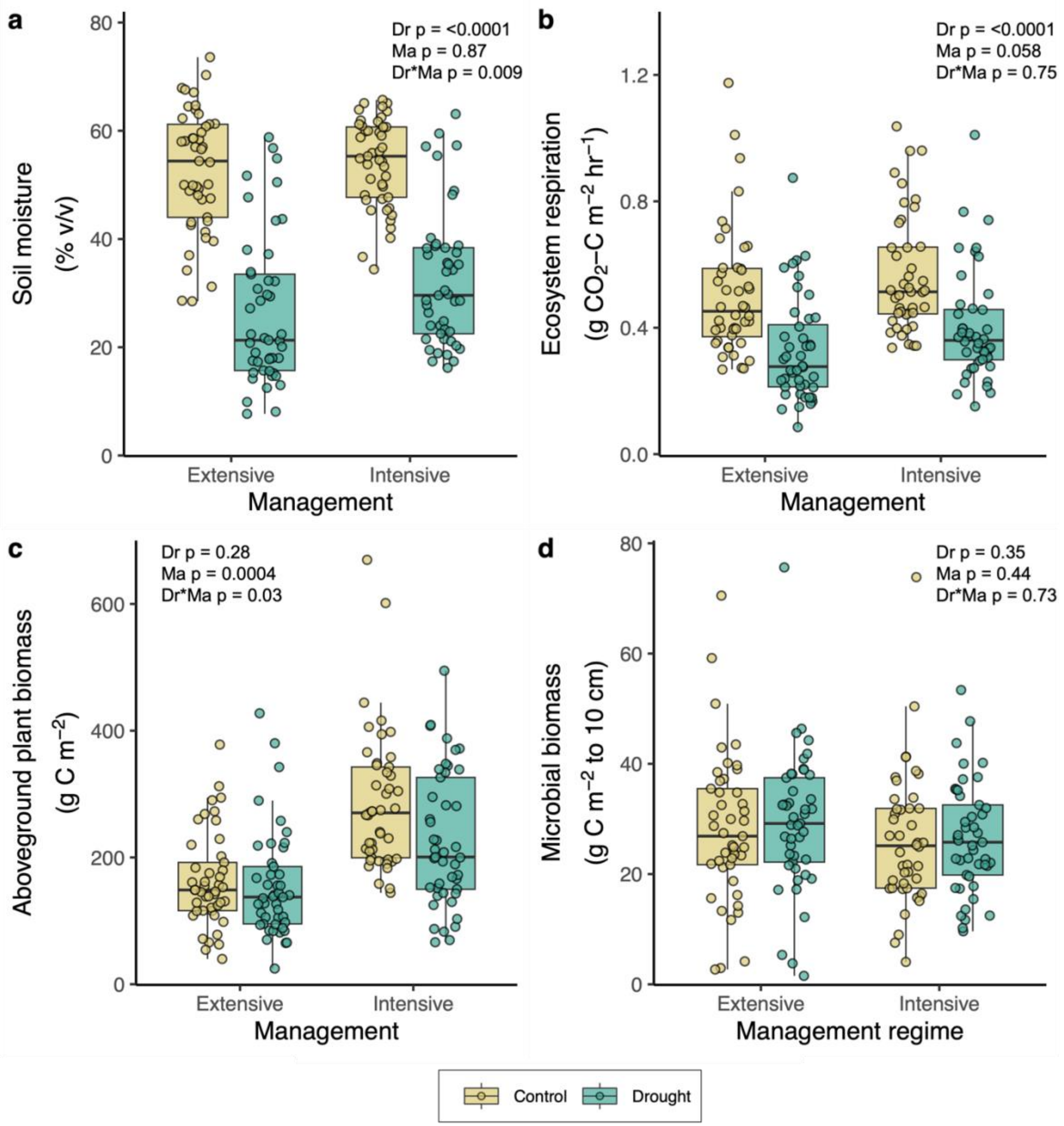

Supplementary Fig. 2. **Effects of drought treatment and grassland management on key ecosystem variables**. Panels show soil moisture (a), ecosystem respiration (b), aboveground plant biomass (c), and microbial biomass (d), measured immediately following removal of drought shelters. P values from linear mixed effect models for drought treatment (Dr), management (Ma), and their interactions (Dr*Ma) are given (n = 180, 175, 180, and 179 experimental plots for panels a, b, c, and d respectively). Boxplots show the median (centre line), first and third quartiles (box limits), and smallest and largest values within 1.5x interquartile range (whiskers), and all datapoints are shown. Source data are provided as a Source Data file.

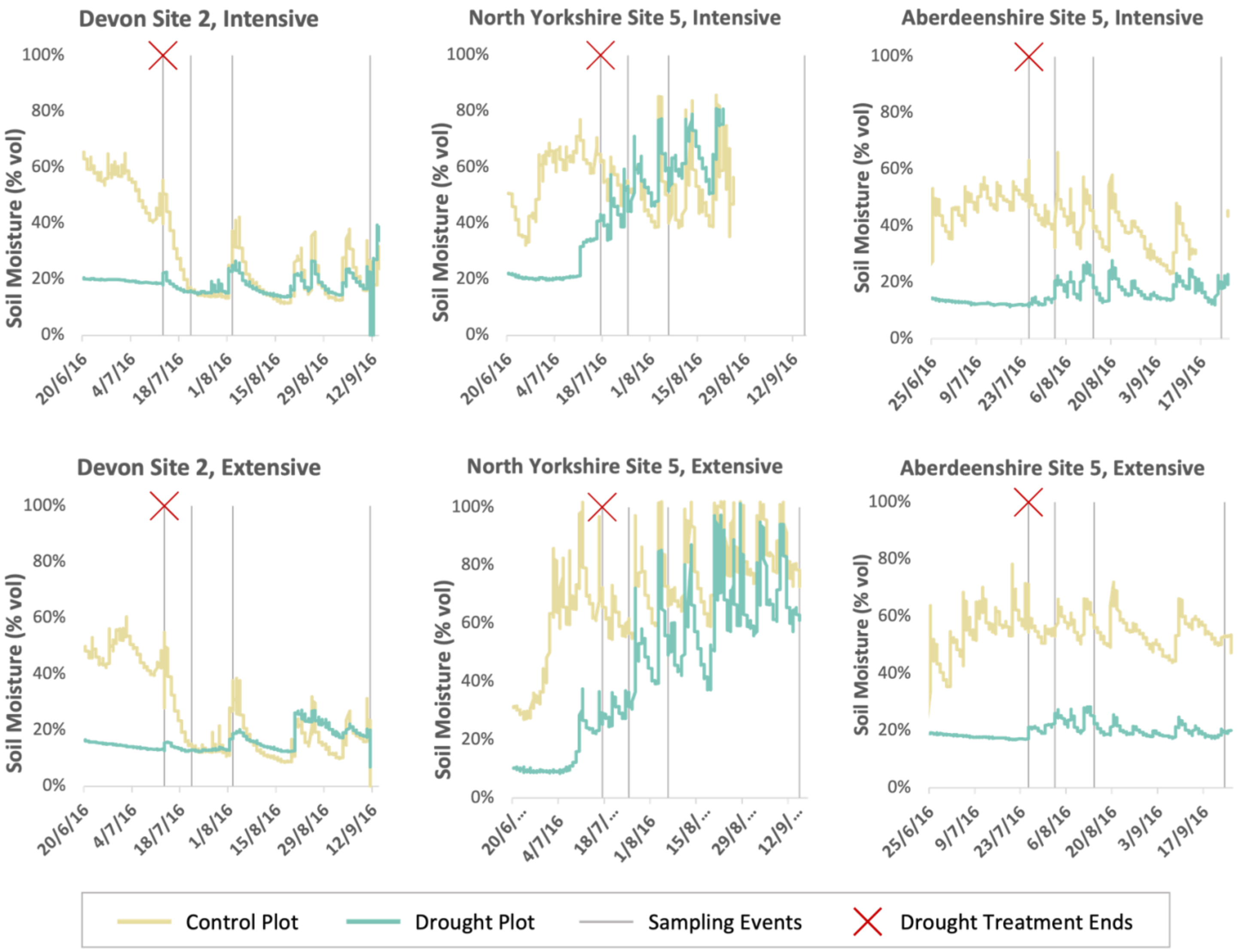

Supplementary Fig. 3. **Moisture probe data.** Moisture probes established in six drought-control pairs show reductions in soil moisture for the duration of the experimental drought period. Four sampling events (gray lines) are shown, but data specific to this study were only collected at the first and last sampling events (immediately following drought and 60 days later).

Supplementary Table 3. **Numbers of dominant taxa.** Count and % of dominant bacterial (16S) and fungal (ITS) OTUs classified under each drought response strategy across all sites. Values in parenthesis are the number or percentage (of total) of opportunistic or sensitive OTUs classified as resilient.

	Bacteria		Fungi	
Drought response strategy	Count	% of dominant OTUs	Count	% of dominant OTUs
Resistant	841	66.3	134	64.1
Opportunistic	243 (192)	19.1 (15.1)	52 (35)	24.8 (16.7)
Sensitive	185 (151)	14.6 (11.9)	23 (15)	11 (7.2)
Total	1269		209	

Supplementary Table 4. **Drought treatment effects on broader bacterial and fungal communities.** Results of perMANOVA analysis (two-sided) of Bray-Curtis dissimilarities using all relative abundance data for a) bacterial (16S) and b) fungal (ITS) genes in relation to drought treatment, region, management and their interactions. Df = degrees of freedom; SumsOfSqs = sums of squares; F.Model = F value by permutation; P values based on 999 permutations (lowest P value possible is 0.001). Asterisks indicate significance at $\alpha = 0.05$. Due to the structure of the model call (stratification at field level), significance levels of terms that do not include drought (gray text) are not accurately represented and should be ignored.

**a)**	Df	SumsOfSqs	MeanSqs	F.Model	R^2	Pr(>F)	
Region	2	3.445	1.72238	23.3038	0.10548	0.001	***
Management	1	2.27	2.27045	30.7192	0.06952	0.001	***
Drought	1	0.271	0.27108	3.6677	0.0083	0.001	***
Field	26	15.865	0.61018	8.2557	0.48578	0.001	***
Region:Drought	2	0.199	0.09935	1.3441	0.00608	0.001	***
Management:Drought	1	0.095	0.09454	1.2791	0.00289	0.019	*
Region:Management:Drought	2	0.167	0.08333	1.1274	0.0051	0.054	.
Residuals	140	10.347	0.07391	0.31684			
Total	175	32.658	1				

**b)**	Df	SumsOfSqs	MeanSqs	F.Model	R^2	Pr(>F)	
Region	2	7.644	3.822	28.3605	0.14127	0.001	***
Management	1	2.492	2.4919	18.4903	0.04605	0.001	***
Drought	1	0.537	0.5367	3.9824	0.00992	0.001	***
Field	26	24.16	0.9292	6.8952	0.44652	0.001	***
Region:Drought	2	0.355	0.1774	1.3163	0.00656	0.014	*
Management:Drought	1	0.182	0.182	1.3504	0.00336	0.033	*
Region:Management:Drought	2	0.275	0.1377	1.022	0.00509	0.4	
Residuals	137	18.463	0.1348	0.34122			
Total	172	54.108	1				

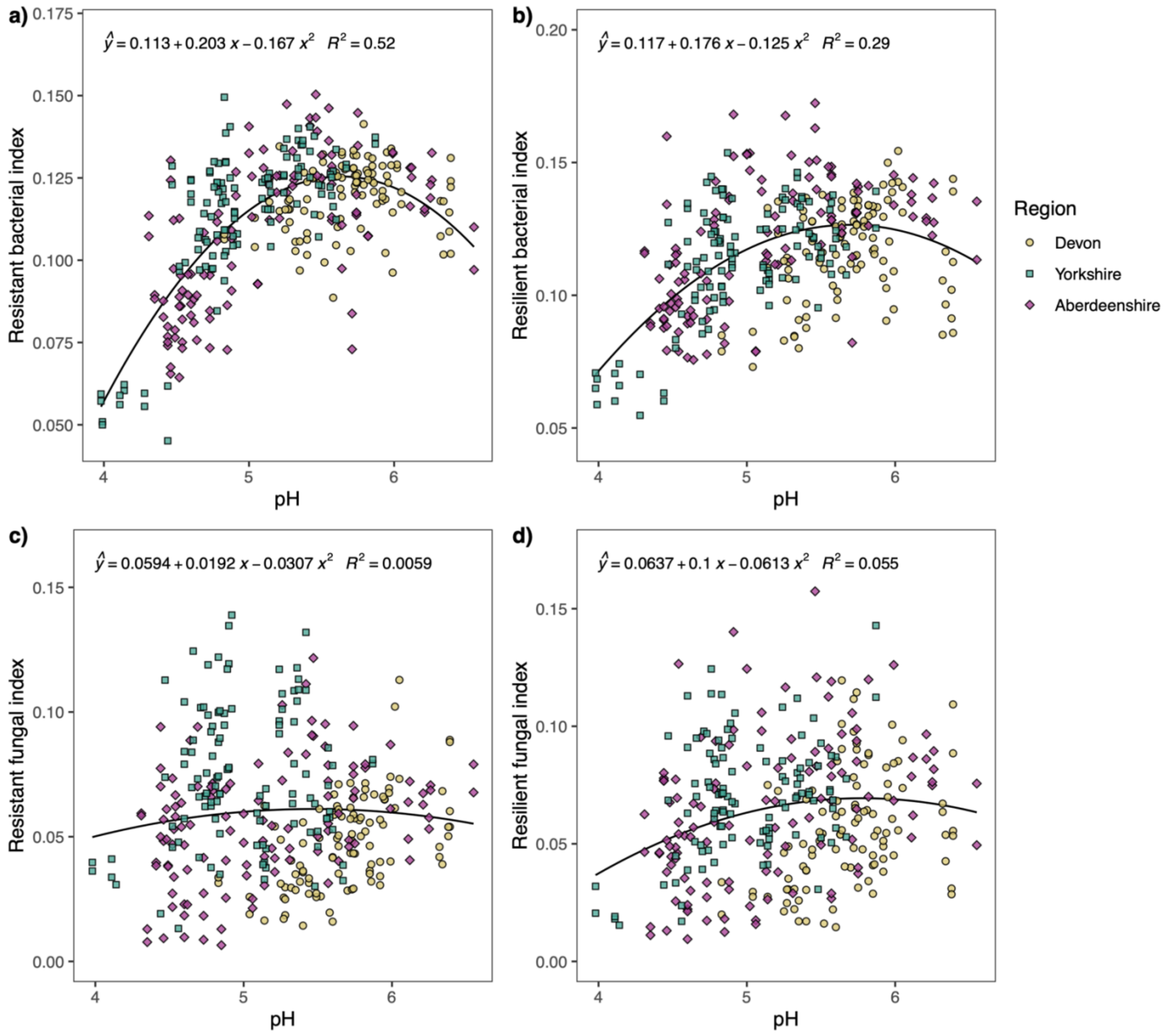

Supplementary Fig. 4. **Effects of pH on dominant soil microbial taxa.** Relationships between soil pH and standardized relative abundances of resistant (a) and resilient (b) bacterial taxa and resistant (c) and resilient (d) fungal taxa across all samples collected in this experiment (n = 180 samples, 90 experimental plots at two time points). Lines are polynomial fits of the data produced with the ggplot2 package in R, with corresponding fit equations and multiple R^2 values.

Supplementary Note 1: Structural Equation Models (SEMs)

Variables

Microbial drought response groupings ("indices" in the main text) in the SEMs are sums of the relative abundances of each OTU standardized relative to their abundances across all samples for each drought response category (opportunistic, sensitive, tolerant).

The "Soil" variable in the SEMs was created by extracting sample values for the first axis of a non-metric multidimensional scaling (NMDS) plot that included total soil carbon, total soil nitrogen, soil texture (% sand), and soil temperature measured at each sampling event. Prior to creating the NMDS, we used a principal coordinates of neighbourhood matrix (PCNM) to account for spatial autocorrelation in the data; spatial autocorrelation accounted for about 16% of spatial variation, while latitude accounted for about 19%. We used the residuals from the spatial model that included the important PCNM vectors (but not latitude) to build the NMDS, and we modeled latitudinal effects explicitly in the SEM. Two soil properties, soil water content (SWC) and pH, were not included in the "soil" variable because they were deemed important to model separately. This was done because both properties are of particular interest for this study: SWC reflects the main effect of the drought treatment relative to the control, and pH is known to be an important control on soil microbial communities and is also managed directly in the intensive fields.

Grassland management was represented as a binary variable (0 = extensive, 1 = intensive). Drought treatment was represented in the same way (0 = control, 1 = drought).

Hypothesized paths

We expected latitude to affect ecosystem properties (soil C and N, soil temperature, pH, soil water content) due to its correlation with climate, a primary control on soil and plant properties. We expected soil properties to influence soil water content via effects of organic matter content on soil water holding capacity[1].

We expected grassland management to affect soil C, and N (components of the "Soil" variable) by influencing plant productivity (inputs to soil C) and nutrient content through fertilization, liming, haying, and seeding. We also expected management to influence pH through liming and fertilization[2], and soil water content via multiple mechanisms (e.g., moisture dynamics via plant biomass and rooting structure).

We expected the relative abundances (normalized as indices) of each group of dominant microbial taxa to be affected by variables that have been shown to control microbial community structure: (1) latitude due to its correlation with climate, (2) soil properties (organic matter content (C and N)[3–5] and temperature, (3) pH[6,7], (4) soil water content[8]. We expected the drought treatment to impact microbial groups separately from soil water content because soil water content was only measured at the individual sampling points, while the drought treatment variable was a broader representation of the treatment itself (potentially reflecting effects aside from soil water content on those particular days). We hypothesized that intensive management would affect dominant microbial groups via the mechanisms described in the main text.

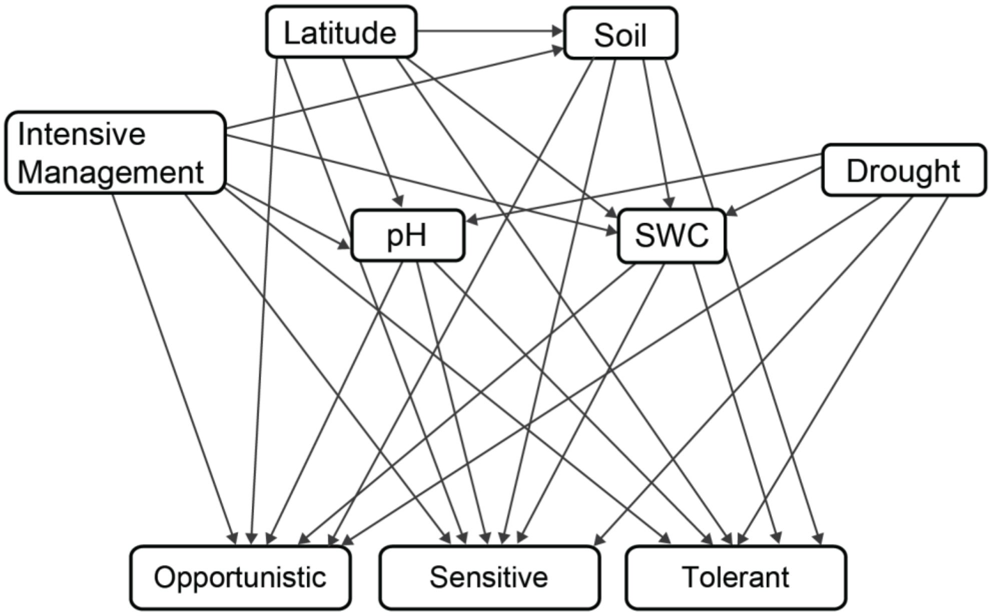

Supplementary Fig. 5. **A priori Structural Equation Model.** Depiction of hypothesized relationships between grassland management, drought treatment, latitude, soil properties, soil moisture, and dominant microbial taxa (grouped according to drought response strategy). See Supplementary Note 1 for additional explanations of variables and paths.

Supplementary Methods

PLFA extraction and analysis

Soil microbial communities were characterised by the extraction of the phospholipid fatty acids (PLFAs), according to Buyer and Sasser[9] and detailed in Chomel et al. [10]. Briefly, fatty acids were extracted from 0.5 g of dry soil in Bligh-Dyer extractant for 2 hr rotating end-over-end. The liquid phase was collected after centrifugation, 1.0 ml chloroform:water (1:1) was added, and the lower phase was siphoned off. Lipids were separated by solid-phase extraction (SPE) in a 96-well plate, each well packed with 100 mg silica (Phenomenex) using chloroform, acetone, and 5:5:1 methanol:chloroform:water. Fatty acids were then transesterified and extracted. Quantities of individual PLFAs were determined by gas chromatography using an Agilent Technologies 7890B gas chromatograph with an Agilent DB-5 ms column. The internal standard 19:0 phosphati-dylcholine (Avanti Polar Lipids, Inc.; Birmingham, AL, USA) added at the beginning of the extraction procedure was used for calculating concentrations. The fatty acids i15:0, a15:0, 15:0, i16:0, 16:1ω7, i17:0, a17:10, 17:0, cy17:0, 18:1ω7, and cy19:0 were used as bacterial markers while the fatty acid 18:2ω6,9 was used as a marker of fungi (Fostergard and Baath, 1996; de Vries et al., 2018). Microbial biomass C was calculated assuming 1mg C is equal to 363.6 nmol [11] bacterial PLFA or 11.8 nmol fungal PLFA [12], and total microbial biomass was calculated as the sum of bacterial and fungal biomass.

Plant-available Nitrogen

Plant-available N was extracted using 5 g field-moist, homogenised soil mixed with 25 mL of 1 M KCl in a 50 mL centrifuge tubes which were shaken horizontally for 1 hour, then left to stand until settled. Extracts were passed through Whatman No. 1 filter paper and analysed colorimetrically on an autoanalyser (AA3 HR AutoAnalyser, Seal Analytical, Soton, UK).

Ecosystem respiration and net ecosystem exchange (NEE)

Gas sampling was performed at each time point (days 0, 8, 20, and 60) to assess ecosystem respiration and net ecosystem exchange (NEE). Gas sampling collars were installed to 6 cm depth (15 cm diameter) in each plot at the same time as the drought treatments were established. All gas measurements were made between 10 am and 2 pm – with a maximum of 20 minutes between measurements of paired fields – using a closed loop technique with a custom chamber (2.3 L) attached to an EGM-4 (PP Systems, Amesbury, MA, USA). For NEE, a transparent chamber was placed on top of the gas sampling collar (sealed with a rubber gasket) and the concentration of CO_2 in the chamber headspace was measured for two minutes. After removing the chamber to vent, respiration was measured in the same way with an opaque cover placed over the chamber to block all incoming light. For all gas measurements, the

first 24 s of data were ignored to account for chamber equilibration and the remaining data was assessed for linearity ($R^2 > 0.7$), with a minimum of 60 s used to calculate an average flux rate.

Aboveground plant biomass

At day 0 only, aboveground plant biomass was collected to assess the impact of the drought on the plant community. In each of the 180 experimental plots, a small quadrat was randomly placed with no edge less than 15 cm inside the plot boundary and all aboveground plant biomass inside the quadrat was cut to within 4 cm of the soil surface. Biomass was collected in paper bags and transported to the laboratory within 24-28 hours where it was dried to constant weight at 40°C, weighed, and stored for further analysis.

Supplementary References

1. Cotrufo, M. F. & Lavallee, J. M. Soil organic matter formation, persistence, and functioning: A synthesis of current understanding to inform its conservation and regeneration. in *Advances in Agronomy* 1–66 (Elsevier Inc., 2021). doi:10.1016/bs.agron.2021.11.002.
2. Tian, D. & Niu, S. A global analysis of soil acidification caused by nitrogen addition. *Environ. Res. Lett.* **10**, 24011–24019 (2015).
3. Ramirez, K. S., Craine, J. M. & Fierer, N. Consistent effects of nitrogen amendments on soil microbial communities and processes across biomes. *Glob. Chang. Biol.* **18**, 1918–1927 (2012).
4. Fierer, N., Bradford, M. A. & Jackson, R. B. Toward an ecological classification of soil bacteria. *Ecology* **88**, 1354–1364 (2007).
5. Rousk, J. *et al.* Soil bacterial and fungal communities across a pH gradient in an arable soil. *ISME J.* **4**, 1340–1351 (2010).
6. Delgado-Baquerizo, M. *et al.* A global atlas of the dominant bacteria found in soil. *Science (80-.).* **359**, 320–325 (2018).
7. Lauber, C. L., Strickland, M. S., Bradford, M. A. & Fierer, N. The influence of soil properties on the structure of bacterial and fungal communities across land-use types. *Soil Biol. Biochem.* **40**, 2407–2415 (2008).
8. Manzoni, S., Schimel, J. P. & Porporato, A. Responses of soil microbial communities to water stress: Results from a meta-analysis. *Ecology* **93**, 930–938 (2012).
9. Buyer, J. S. & Sasser, M. High throughput phospholipid fatty acid analysis of soils. *Appl. Soil Ecol.* **61**, 127–130 (2012).
10. Chomel, M. *et al.* Drought decreases incorporation of recent plant photosynthate into soil food webs regardless of their trophic complexity. *Glob. Chang. Biol.* **25**, 3549–3561 (2019).
11. Frostegård, A. & Bååth, E. The use of phospholipid fatty acid analysis to estimate bacterial and fungal biomass in soil. *Biol. Fertil. Soils* **22**, 59–65 (1996).
12. Klamer, M. & Bååth, E. Estimation of conversion factors for fungal biomass determination in compost using ergosterol and PLFA 18:2ω6,9. *Soil Biol. Biochem.* **36**, 57–65 (2004).
