# Supplementary material for: Land management shapes drought responses of dominant soil microbial taxa across grasslands
Source: Nat Commun. 2024 Jan 2;15:29. doi: 10.1038/s41467-023-43864-1 (PMC10762234; doi:10.1038/s41467-023-43864-1)
Supplement: Supplementary file 2 — Reporting Summary [file 41467_2023_43864_MOESM2_ESM.pdf]

nature portfolio

Corresponding author(s): Dr. Jocelyn M Lavallee

Last updated by author(s): Sep 17, 2023

Reporting Summary

Nature Portfolio wishes to improve the reproducibility of the work that we publish. This form provides structure for consistency and transparency in reporting. For further information on Nature Portfolio policies, see our Editorial Policies and the Editorial Policy Checklist.

Statistics

For all statistical analyses, confirm that the following items are present in the figure legend, table legend, main text, or Methods section.

n/a	Confirmed	
☐	☒	The exact sample size (*n*) for each experimental group/condition, given as a discrete number and unit of measurement
☐	☒	A statement on whether measurements were taken from distinct samples or whether the same sample was measured repeatedly
☐	☒	The statistical test(s) used AND whether they are one- or two-sided<br>*Only common tests should be described solely by name; describe more complex techniques in the Methods section.*
☐	☒	A description of all covariates tested
☐	☒	A description of any assumptions or corrections, such as tests of normality and adjustment for multiple comparisons
☐	☒	A full description of the statistical parameters including central tendency (e.g. means) or other basic estimates (e.g. regression coefficient) AND variation (e.g. standard deviation) or associated estimates of uncertainty (e.g. confidence intervals)
☐	☒	For null hypothesis testing, the test statistic (e.g. *F*, *t*, *r*) with confidence intervals, effect sizes, degrees of freedom and *P* value noted<br>*Give P values as exact values whenever suitable.*
☒	☐	For Bayesian analysis, information on the choice of priors and Markov chain Monte Carlo settings
☐	☒	For hierarchical and complex designs, identification of the appropriate level for tests and full reporting of outcomes
☒	☐	Estimates of effect sizes (e.g. Cohen's *d*, Pearson's *r*), indicating how they were calculated

Our web collection on statistics for biologists contains articles on many of the points above.

Software and code

Policy information about availability of computer code

Data collection	No software was used for data collection.
Data analysis	R version 4.0.2, with packages:<br>nlme version 3.1-148<br>glmmTMB version 1.1.5<br>DHARMa version 0.4.6<br>lavaan version 0.6-12<br><br>PEAR (http://www.exelixis-lab.org/web/software/pear)<br>FASTX (http://hannonlab.cshl.edu/fastx_toolkit/)<br>bbTOOLS (https://jgi.doe.gov/data-and-tools/software-tools/bbtools/)<br>PIPITS (https://github.com/hsgweon/pipits)<br><br>All code is available from GitHub [DOI: 10.5281/zenodo.10121576]

For manuscripts utilizing custom algorithms or software that are central to the research but not yet described in published literature, software must be made available to editors and reviewers. We strongly encourage code deposition in a community repository (e.g. GitHub). See the Nature Portfolio guidelines for submitting code & software for further information.

Data

Policy information about availability of data

All manuscripts must include a data availability statement. This statement should provide the following information, where applicable:

- Accession codes, unique identifiers, or web links for publicly available datasets
- A description of any restrictions on data availability
- For clinical datasets or third party data, please ensure that the statement adheres to our policy

The sequence data generated in this study have been deposited in the EMBL Nucleotide Sequence Database (ENA) under accession code PRJEB63076 [https://www.ebi.ac.uk/ena/browser/search]. All other raw data generated in this study have been deposited in the figshare repository [DOI: https://doi.org/10.6084/m9.figshare.22731971] and on GitHub [DOI:10.5281/zenodo.10121576].

Research involving human participants, their data, or biological material

Policy information about studies with human participants or human data. See also policy information about sex, gender (identity/presentation), and sexual orientation and race, ethnicity and racism.

Reporting on sex and gender	N/A
Reporting on race, ethnicity, or other socially relevant groupings	N/A
Population characteristics	N/A
Recruitment	N/A
Ethics oversight	N/A

Note that full information on the approval of the study protocol must also be provided in the manuscript.

Field-specific reporting

Please select the one below that is the best fit for your research. If you are not sure, read the appropriate sections before making your selection.

☐ Life sciences ☐ Behavioural & social sciences ☒ Ecological, evolutionary & environmental sciences

For a reference copy of the document with all sections, see nature.com/documents/nr-reporting-summary-flat.pdf

Ecological, evolutionary & environmental sciences study design

All studies must disclose on these points even when the disclosure is negative.

Study description	This study was carried out on working farms with contrasting pairs of grasslands under different management intensities (extensive and intensive). The design was a hierarchical nested design. At the top level, there were three regions (Aberdeenshire, North Yorkshire, Devon). Within each region, we identified 5 sites that each had a pair of grasslands with different management (one extensive, one intensive). In each grassland, we constructed three plots, each with a paired treatment (rain shelter) and control (no shelter). In all, there were 3 regions x 5 sites x 2 managements x 3 plots x 2 treatment levels (drought, control) for 180 total plots. Each plot was sampled at two time points: immediately following drought, and after a 60-day post drought recovery period, for 360 total samples.
Research sample	The research sample consists of 360 soil samples taken at two time points from the same experimental plots (180 plots). The samples come from working farms across three regions with broad ranges in climate, soil properties, plant species, and productivity. The sample was designed to broadly represent managed UK grasslands by including working farms under a variety of environmental conditions, within the allowance of available budget and personnel resources. The sample follows a factorial design with three regions, each with five sites, each of which include of two fields with contrasting management (intensive, extensive), two drought treatments (ambient, drought), and three field replicates.
Sampling strategy	Sample size calculations were not performed. We designed the sampling scheme to capture variability across managed UK grasslands based on in-depth team knowledge of managed grasslands in the UK, but constrained by logistical feasibility (funding, number of staff, time required, spatial separation and travel time between sites, etc.). This number of sites and samples was chosen to be broadly representative of the range of conditions across managed UK grasslands, and also to be feasible to set up experimental treatments and sample at multiple time points given available resources.
Data collection	Some data were collected in the field, and some in the laboratory. Relevant methodological details are provided with the manuscript. All data were collected and recorded by the authors using pen and paper or Microsoft Excel.

Timing and spatial scale	Field measurements and soil samples were collected 60 days apart. The first sampling event occurred following the drought treatment period, in July of 2016. Sampling events were spaced several days apart between regions, so that Devon sites were sampled first (July 12-14), then North Yorkshire sites (July 17-19), then Aberdeenshire sites (July 25-27) to allow the field team time to process samples and travel between sites, and considering differences in climate and phenology from south to north. (Note: the drought treatment set-up followed the same schedule, so that the drought treatment length was the same in each region.) The second sampling followed the same schedule but 60 days later, in September 2016. This post-drought recovery period was chosen as an appropriate length to allow responses in microbial and plant communities based on past experience of the research team, while also constraining sampling to the more productive summer period and avoiding colder temperatures and lower productivity of the autumn season.<br>The spatial scale of the data at the lowest level is the plot level (~3 square meters), which were clustered in areas of ~ 15 square meters within each field. Fields varied in size but tended to be one-two hectares or less. The spatial scale of the sites (paired fields) and regions is shown in a map in the Supplemental Information.
Data exclusions	No data were excluded from the analysis.
Reproducibility	Given that this was a field experiment, it is not directly reproducible. However, a similar manipulative drought treatment could be carried out elsewhere and we have provided the methodological details necessary to carry out similar experiments.
Randomization	We accounted for important covariates or random effects in models to account for the spatial nature of our experimental design. For the Structural Equation Models, within-field replicates were averaged to account for their lack of independence. In all other modeling using mixed effects models, the hierarchical experimental design was accounted for using nested random effects as descried in the methods section.
Blinding	Blinding in the traditional sense was not relevant to this study as we did not have patients or human subjects. We did, however, minimize any potential bias during laboratory analysis by using random numbering schemes on all laboratory samples (rather than label identifiers with location and treatment information).

Did the study involve field work? ☒ Yes ☐ No

Field work, collection and transport

Field conditions	Fieldwork was carried out on multiple days during July and September of 2016. Prevailing weather conditions varied throughout the sampling period, though recorded soil temperatures did not vary by more than 9 degrees C within each region.
Location	Sampling locations are as follows (latitude, longitude in decimal degrees):<br>50.6816698, -3.7814391<br>50.6814659, -3.7813161<br>50.7616704, -3.8509559<br>50.7615313, -3.8499282<br>50.7738512, -3.9106843<br>50.7784521, -3.9089115<br>50.9260446, -4.3556594<br>50.9264818, -4.3553925<br>50.9509268, -4.2970946<br>50.9521943, -4.2967606<br>56.6988439, -3.4961479<br>56.698038, -3.4955116<br>56.7381171, -3.4004376<br>56.7377992, -3.4003547<br>56.7486386, -3.4025011<br>56.7487218, -3.4024126<br>56.9002628, -2.5353148<br>56.900453, -2.5354773<br>56.9025156, -2.5636446<br>56.9024364, -2.5635579<br>54.2127352, -2.390683<br>54.2126858, -2.3909822<br>54.2535803, -2.3183043<br>54.2531371, -2.3188667<br>54.2672126, -2.2658868<br>54.2671709, -2.2656828<br>54.3267934, -2.2886191<br>54.3269054, -2.2883019<br>54.3396547, -2.3203264<br>54.3397265, -2.3208405
Access & import/export	Given the sampling and experimental work was done on managed, working farms and samples were processed locally, special precautions and permits were not required.

Disturbance	This study did not cause any significant disturbance to the soils or fields sampled. All activities were discussed with and approved by the farmers and landowners prior to execution.

Reporting for specific materials, systems and methods

We require information from authors about some types of materials, experimental systems and methods used in many studies. Here, indicate whether each material, system or method listed is relevant to your study. If you are not sure if a list item applies to your research, read the appropriate section before selecting a response.

Materials & experimental systems

n/a	Involved in the study
☒	☐ Antibodies
☒	☐ Eukaryotic cell lines
☒	☐ Palaeontology and archaeology
☒	☐ Animals and other organisms
☒	☐ Clinical data
☒	☐ Dual use research of concern
☒	☐ Plants

Methods

n/a	Involved in the study
☒	☐ ChIP-seq
☒	☐ Flow cytometry
☒	☐ MRI-based neuroimaging
